# A Case of Hernia Proven by Post-Mortem to Contain Bile and Pus

**Published:** 1875-05-15

**Authors:** U. P. Stair

**Affiliations:** Black Earth, Wis.


### A CASE OF SUPPOSED HERNIA PROVEN BY POST MORTEM TO CONTAIN BILE AND PUS.

BY U. P. STAIR, M.D., BLACK EARTH, WIS.

H. M. W. died on April 8th, 1875, of what was diagnosed to be cancer of the stomach. He had, during the latter years of his life, a small, fluctuating tumor in the epigastric region, about half way between the umbilicus and the ensiform cartilage, This enlargement was about the size of a large almond, and at times it gave to the touch a sensation as though it contained air mixed with liquids. Several physicians who examined it pronounced it a hernia, but what its contents were remained a mystery. An autopsy was held on the 10th ult. There were present Drs. Field, of Sun Prairie, Wis., U. P. Stair, of Black Earth, T. F. Stair, of Mazomanie, and J. B. Stair.

Two incisions were made, following the lower margin of the ribs, beginning at the epigastrium, intersected by another drawn from the latter point to the pubis. When the two triangular flaps were thrown back so as to disclose the stomach, it appeared to be a complete mass of cancerous growth. Upon closer inspection it was found to extend from the pyloric orifice of the stomach around the greater curvature to the spleen, involving that organ with the pancreas, duodenum, and passing through the coats of the stomach, thus uniting these organs into one combination of carcinomatous formation. The stomach was, of course, very much contracted in size by the deposit in its walls, especially throughout the greater curvature. The pylorus was almost entirely closed. From the peculiar character and consistence of the tumor, it was decided to be a cancer of the colloid variety.

The gall-bladder was largely distended, showing that there was obstruction to the free passage of bile. The *round ligament* (umbilical vein in the fœtus) which extends from the notch in the anterior border of the liver to the umbilicus, was found to be strongly adherent to the abdominal tissues at the site of the tumor above mentioned. It was also pervious and filled with bile. The adipose tissue beneath the cuticle in this situation was found largely infiltrated with biliary pigment, a condition not detected upon any other part of the body, showing that it must have been communicated in *some* way through the parietes of the abdomen in that circumscribed locality.

It might be well to state that one point of diagnosis, in the supposition that it was hernia, was the peculiar crepitation caused by the presence of air and liquids. Dissection proved that there was, indeed, liquid contained therein. Air was doubtless present as well. Where did it come from? That the round ligament was hollow and pervious was plainly revealed by section, and also that it contained bile and pus, as did the sac of the supposed hernia. Now could it be possible that air could arrive at and occupy the sac with these liquids from the duodenum by means of the gall-ducts?

Circumstances prevented minute dissection of the tissues in the region of the gall bladder and ducts.

